# A miRNA-HERC4 pathway promotes breast tumorigenesis by inactivating tumor suppressor LATS1

**DOI:** 10.1007/s13238-019-0607-2

**Published:** 2019-02-01

**Authors:** Youqin Xu, Kaiyuan Ji, Meng Wu, Bingtao Hao, Kai-tai Yao, Yang Xu

**Affiliations:** 1 Guangdong Provincial Key laboratory of Tumor Immunotherapy, School of Basic Medical Sciences, Cancer Research Institute, Southern Medical University, Guangzhou, 510632 China; 2 Division of Biological Sciences, University of California, San Diego, 9500 Gilman Drive, La Jolla, CA 92093 USA

**Keywords:** E3 ligase, tumorigenesis, ubiquitination, tumor suppressor, miRNA

## Abstract

The E3 ligase HERC4 is overexpressed in human breast cancer and its expression levels correlated with the prognosis of breast cancer patients. However, the roles of HERC4 in mammary tumorigenesis remain unclear. Here we demonstrate that the knockdown of HERC4 in human breast cancer cells dramatically suppressed their proliferation, survival, migration, and tumor growth *in vivo*, while the overexpression of HERC4 promoted their aggressive tumorigenic activities. HERC4 is a new E3 ligase for the tumor suppressor LATS1 and destabilizes LATS1 by promoting the ubiquitination of LATS1. miRNA-136-5p and miRNA-1285-5p, expression of which is decreased in human breast cancers and is inversely correlated with the prognosis of breast cancer patients, are directly involved in suppressing the expression of HERC4. In summary, we discover a miRNA-HERC4-LATS1 pathway that plays important roles in the pathogenesis of breast cancer and represents new therapeutic targets for human breast cancer.

**Electronic supplementary material:**

The online version of this article (10.1007/s13238-019-0607-2) contains supplementary material, which is available to authorized users.

## Introduction

Breast cancer is one of the most deadly cancers worldwide with increasing frequency and mortality rates (Dubey et al., 2015). The mammary tumorigenesis is a multistep process involving various genetic and epigenetic alterations, leading to the activation of various oncoproteins or inactivation of tumor suppressors (Duffy et al., 2000; Weidle et al., 2018). For example, several oncogenic receptors such as estrogen receptor (ER), progesterone receptor (PR) and human epidermal growth factor type 2 receptor (HER2), have been identified as the key regulators of mammary tumorigenesis (Patani et al., 2013; Mayer et al., 2017). Due to its genetic and epigenetic heterogeneity, breast cancers of advanced stages remain very difficult to treat. Therefore, it is critical to elucidate the pathways that drive breast tumorigenesis to identify new and more effective therapeutic targets.

The Hippo pathway plays critical roles in controlling the organ and tissue homeostasis by modulating cellular proliferation and apoptosis, and is deregulated in various human cancers (Pfleger, 2017). The Hippo pathway is a tumor suppressor pathway consisting of multiple proteins, including MST1/2, MOB1 and large tumor suppressor 1/2 (LATS1/2) (Furth and Aylon, 2017; Kulaberoglu et al., 2017). LATS1/2 kinases phosphorylate YAP and TAZ to inactivate these oncogenic proteins (Furth and Aylon, 2017). In addition, LATS kinases can suppress breast cancer by promoting the ubiquitination and degradation of ERα as well as inhibiting the differentiation of bipotent luminal progenitors, a proposed cellular origin of human breast cancers (Britschgi et al., 2017).

Ubiquitin-proteasome system (UPS) regulates the dynamic change of protein levels of intracellular proteins (Hershko, 2005) and plays important roles in cancer development (Micel et al., 2013; Mofers et al., 2017). The six members of the HERC family proteins contain both HECT and RCC-1-like domains (Mitsui et al., 1999). The HECT domain can function as a ubiquitin/ubiquitin-like (UBL) protein ligase by ligating polyubiquitin chains to proteins for recognition and degradation via the proteasome (Rotin and Kumar, 2009). Accumulating data demonstrate that HERC family proteins are functionally linked to malignant tumor development. For example, HERC1, the first identified member of the HERC family protein, is a key E3 ligase of MutS homolog 2 (MSH2), a DNA mismatch repair enzyme that plays a pivotal role in maintaining genomic integrity (Diouf et al., 2011). The expression levels of HERC2, which is involved in DNA double-strand break (DSB) repair, are positively correlated with the poor prognosis of non-small-cell lung cancer (Bonanno et al., 2016).

Another member of the HERC family proteins, HERC4, plays important roles in spermatogenesis and male fertility (Rodriguez and Stewart, 2007). HERC4 is overexpressed in several types of cancer such as lung cancer and hepatocellular carcinoma (Zeng et al., 2015; Zheng et al., 2017). The overexpression of HERC4 is correlated with the poor prognosis of breast cancer patients (Zhou et al., 2013). Here we demonstrate that HERC4 promotes breast cancer progression by destabilizing tumor suppressor LATS1. In addition, we identified the upstream tumor suppressive miRNAs that suppress breast tumorigenesis by inhibiting the expression of HERC4 in human breast cancer cells.

## Results

### HERC4 promoted tumorigenesis of breast cancer cells

**Figure 1:**
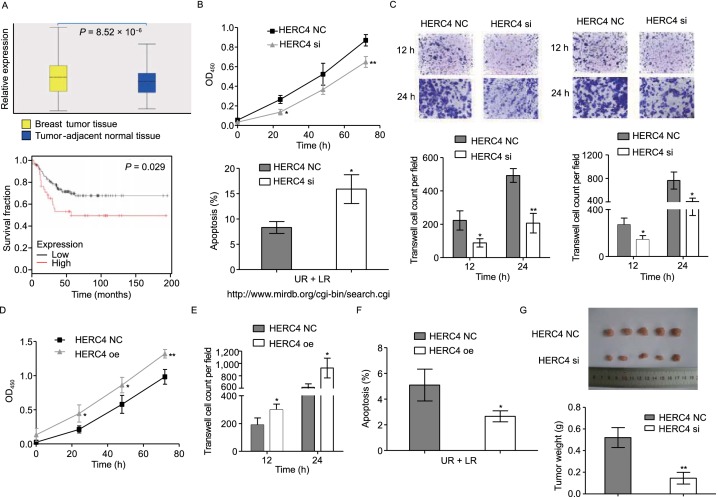
**HERC4 promotes tumorigenesis of breast cancer cells**. (A) The comparison of the expression of *HERC4* (top panel) in human breast cancers (*n* = 602) and tumor-adjacent normal tissues (*n* = 508). Log-rank (Mantel Cox) survival test of breast cancer patients (bottom panel) based on the levels of HERC4 mRNA (low expression *n* = 120, high expression *n* = 41). *P* values are indicated. (B) The proliferation (top panel) and apoptosis (bottom panel) of MCF-7 cells before and after HERC4 knockdown. The cell number was determined with CCK-8 assay. Upper right (UR, PI^+^Annexin^+^) and Lower right (LR, PI^−^Annexin^+^) were counted as apoptotic cells. *n* = 3. Data are represented as mean ± standard deviation (s.d.). (C) Knockdown of HERC4 inhibited the migration (left panel) and invasion (right panel) of MCF-7 cells using a transwell assay. *n* = 3. Data are represented as mean ± s.d. (D–F) The overexpression of HERC4 (HERC4 oe) in MCF-7 cells promoted the proliferation (D), migration (E), and survival (F) of breast cancer cells. *n* = 3. Data are represented as mean ± s.d. (G) The knockdown of HERC4 suppressed the tumor growth of MCF-7 cells in nude mice. *n* = 5. *P < 0.05, **P < 0.01

Using the expression data of *HERC4* in breast tumors and tumor-adjacent normal tissues in the database (GSE 93601), we confirmed that *HERC4* was overexpressed in breast cancers (Fig. 1A, top panel). In addition, the expression levels of *HERC4* were inversely correlated with the prognosis of human breast cancer patients (Fig. 1A, bottom panel). These data support the notion that HERC4 promotes breast tumorigenesis. To investigate the roles of HERC4 in breast tumorigenesis, we examined the expression levels of HERC4 mRNA and protein in various breast cancer cell lines. When compared to the normal breast epithelial cell line MCF-10A, the expression levels of HERC4 mRNA and protein were increased in breast cancer cell lines such as MDA-MB-231 and MCF-7 cells (Fig. S1A and S1B). Therefore, we examined the roles of HERC4 in these breast cancer cells by knockdown or overexpression of HERC4 (Fig. S1C–F). The silencing of HERC4 decreased the cellular proliferation and survival of MCF-7 cells (Fig. 1B). Using transwell assays, we further showed that the silencing of HERC4 inhibited the migration of MCF7 cells (Fig. 1C). These data indicate that HERC4 promotes various aspects of tumorigenesis of breast cancer cells. Consistent with this conclusion, the overexpression of HERC4 in MCF7 cells promoted the proliferation, migration and survival of MCF7 cells (Fig. 1D–F). In further support of an important role of HERC4 in the tumorigenesis of breast cancer cells, the knockdown of HERC4 in MCF7 cells significantly reduced the growth of tumors formed by MCF7 cells in immunodeficient mice (Fig. 1G). Consistent data were obtained using another breast cancer cell line MDA-MB-231 cells (Fig. S2). Since HERC4 is also frequently overexpressed in human lung cancers, we also studied the roles of HERC4 in human lung cancer cell line A549. The knockdown of HERC4 in A549 lung cancer cells also suppressed their cellular proliferation, migration and survival (Fig. S3). These findings demonstrate that HERC4 is important for promoting tumorigenesis.

### HERC4 destabilizes tumor suppressor LATS1

**Figure 2:**
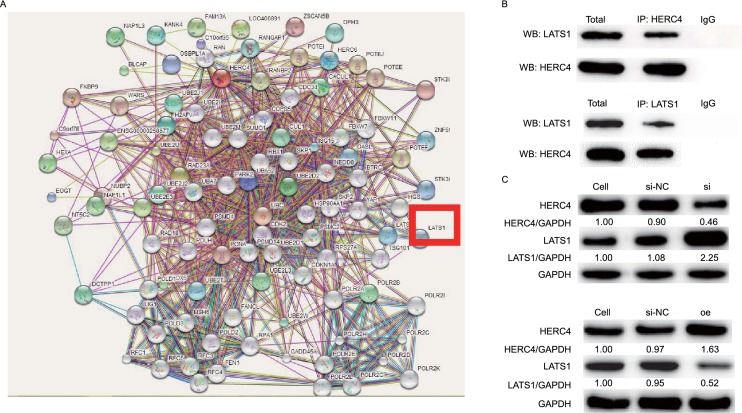
**HERC4 interacted with LATS1**. (A) STRING analysis predicted a network of 271 proteins that might interact with HERC4. LATS1 is indicated with a red box. (B) HERC4 was confirmed to interact with LATS1 in MCF-7 cells with Co-IP assay. (C) The protein levels of LATS1 were inversely correlated with the protein levels of HERC4 in MCF-7. The protein levels of HERC4 were regulated with the knockdown (si) or overexpression (oe) of HERC4 in MCF-7 cells

As an E3 ligase, we predicted that HERC4 likely functioned by regulating the stability of other proteins involved in tumorigenesis. Therefore, to understand the mechanisms how HERC4 promotes breast tumorigenesis, we used STRING Protein-Protein Interaction database to predict the proteins that might interact with HERC4 (Fig. 2A). One identified candidate was the tumor suppressor LATS1 known to suppress breast tumorigenesis. Using co-immunoprecipitation (CO-IP) assay, we confirmed the interaction between HERC4 and LATS1 in breast cancer cells (Fig. 2B). In addition, the protein levels of HERC4 were inversely correlated with the protein levels of LATS1 in breast cancer cells, supporting the notion that HERC4 negatively regulates the protein levels of LATS1 (Fig. 2C).

**Figure 3:**
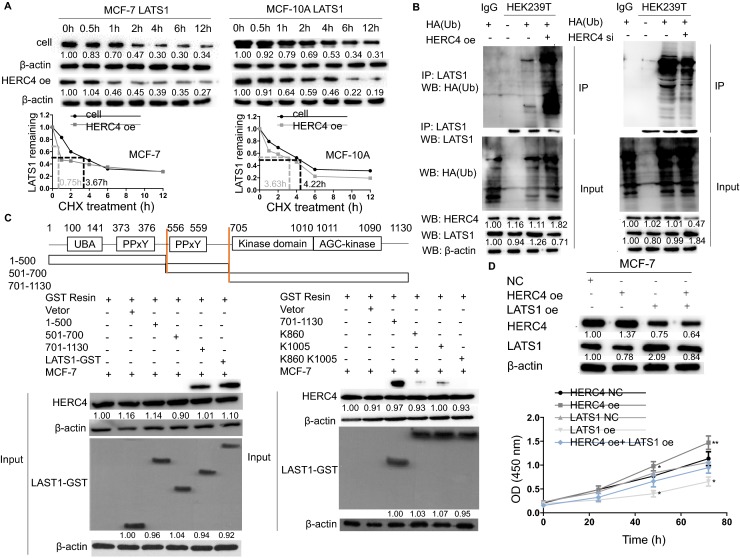
**HERC4 destabilized tumor suppressor LATS1**. (A) The overexpression of HERC4 (HERC4 oe) in MCF-7 breast cancer cells and MCF-10A normal mammary epithelial cells reduced the stability of LATS1. (B) The protein levels of HERC4 were inversely correlated with the ubiquitination levels of LATS1. The HA tagged ubiquitin was expressed in 293 cells with overexpression (oe) or knockdown (si) of HERC4. LATS1 was immunoprecipitated and its ubiquitination levels determined with anti-HA antibody (top panel). As an internal control, the protein levels in the input were determined by Western blot (bottom panel). (C) HERC4 was bound to the C-terminus of the LATS1 (left panel). HERC4 was co-expressed with GST-LATS1 or GST-LATS1 deletion mutants, and their interaction verified by GST pull-down assay (left panel). K860R and K1005R mutants of GST-LATS1 failed to pull down HERC4 (right panel). (D) The overexpression of HERC4 partially rescued the proliferative defects induced by LATS1 overexpression. *n* = 3. *P < 0.05, **P < 0.01

To test whether HERC4 can destabilize LATS1, the overexpression of HERC4 significantly reduced the half-life of LATS1 in both normal breast epithelial cells and breast cancer cells, indicating that HERC4 destabilizes LATS1 (Fig. 3A). In support of the hypothesis that HERC4 could serve as an E3 ligase for LATS1, the overexpression of HERC4 increased the ubiquitination of LATS1 and the knockdown of HERC4 reduced the ubiquitination of LATS1 (Fig. 3B). To identify the region of LATS1 that is involved in interacting with HERC4, we examined the interaction between HERC4 and the three deletion mutants of LATS1, and found that HERC4 interacted with the C-terminus (aa 701–1,130) of LATS1 that contained two putative ubiquitination sites K860 and K1005 (Fig. 3C). The overexpression of LATS1 reversed the tumorigenic activities induced by the overexpression of HERC4 in breast cancer cells (Fig. 3D). Therefore, HERC4 promotes the breast tumorigenesis by destabilizing LATS1.

**Figure 4:**
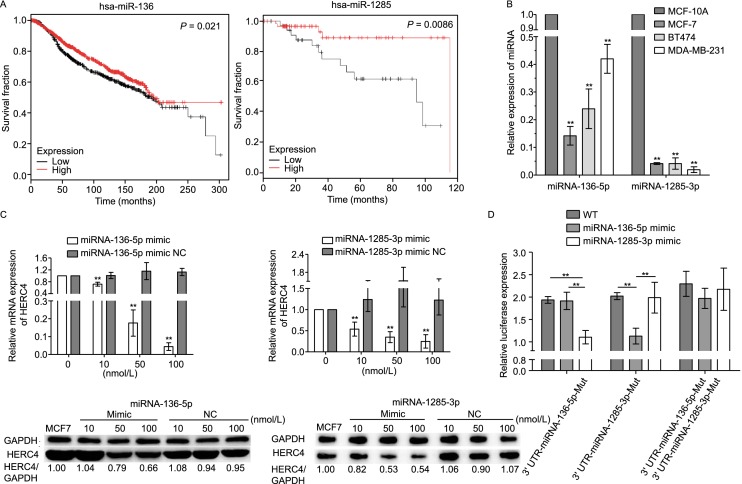
**miR-1285-3p and miR-136-5p directly suppressed the expression of HERC4**. (A) Log-rank (Mantel Cox) survival test of breast cancer patients based on the levels of hsa-miR-136 (left panel: low expression *n* = 536, high expression *n* = 726) and hsa-miR-1285 (right panel: low expression *n* = 37, high expression *n* = 60). (B) The levels of miRNA-136-5p and miRNA-1285-3p in various breast cancer cells and normal breast epithelial cells. *n* = 3. Data are represented as mean ± s.d. (C) The overexpression of miRNA-136-5p or miRNA-1285-3p reduced the levels of HERC4 mRNA (top panel, *n* = 3) and protein (bottom panel). Data are represented as mean ± s.d. (D) miRNA-136-5p and miRNA-1285-3p target the predicted sites within the 3′-UTR of the HERC4 mRNA. The mutation of the predicted target site reversed the inhibitory effects of miRNA-136-5p and miRNA-1285-3p. *n* = 3. *P < 0.05, **P < 0.01

### miR-1285-3p and miR-136-5p negatively regulate the expression of HERC4

miRNA plays important roles in tumor suppression or oncogenesis (Campos-Parra et al., 2017; Rupaimoole and Slack, 2017). Considering the importance of HERC4 in promoting breast tumorigenesis, we used miRDB prediction program (http://www.mirdb.org/cgi-bin/search.cgi) and GEO datasets (GSE42072) to predict miRNAs that might target HERC4 mRNA. Based on the predicted results, two miRNAs, miR-1285-3p and miR-136-5p, were identified as potential regulators of HERC4a mRNA. Consistent with previous findings that miR-136 was downregulated in triple negative breast cancers (Yan et al., 2016), we found that the expression levels of miR-1285-3p and miR-136-5p were higher in tumor-adjacent normal tissues than in breast tumor tissues (Fig. S4B). In addition, the expression levels of miR-1285-3p and miR-136-5p in breast cancers were inversely correlated with the prognosis of breast cancer patients (Fig. 4A).

To determine the impact of miR-1285-3p and miR-136-5p on the expression of *HERC4*, we used miRNA mimics to demonstrate that the expression levels of these two miRNAs were inversely correlated with the expression levels of HERC4, supporting the notion that these two miRNAs might directly target HERC4 mRNA (Fig. 4B–D and Fig. S4C–D). By mutating the predicted target sites of these miRNAs in the 3′ UTR of HERC4 mRNA, we confirmed that miR-1285-3p and miR-136-5p regulated the levels of HERC4 mRNA by targeting the predicted sites within the 3′ UTR of HERC4 (Figs. 4E and S4).

**Figure 5:**
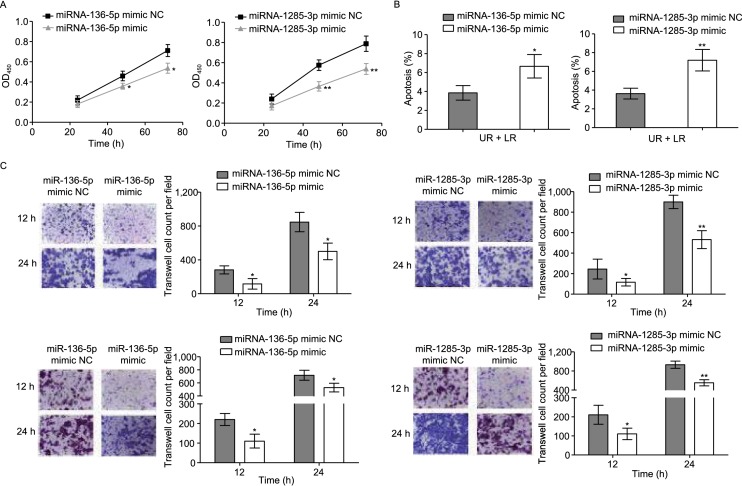
**The overexpression of miRNA-136-5p or miRNA-1285-3p in cells suppressed their tumorigenic activities**. (A) The overexpression of miRNA-136-5p (left panel) or miRNA-1285-3p (right panel) reduced the proliferation of MCF7 cells. *n* = 3. Data are represented as mean ± s.d. (B) The overexpression of miRNA-136-5p (left panel) or miRNA-1285-3p (right panel) induced the apoptosis of MCF7 cells. The cells were stained with propidium iodide (PI) and FITC-conjugated Annexin V. Upper right (UR, PI^+^Annexin^+^) and Lower right (LR, PI^−^Annexin^+^) were counted as apoptotic cells. *n* = 3. Data are represented as mean ± s.d. (C) The overexpression of miRNA-136-5p (left panel) or miRNA-1285-3p (right panel) reduced the migration (top panel) and invasion (bottom panel) of MCF7 cells. *n* = 3. Data are represented as mean ± s.d. *P < 0.05, **P <0.01

Since miR-1285-3p and miR-136-5p suppress HERC4 expression in breast cancer cells, we predicted that these miRNAs could have the same tumor suppressive effects as the silencing of HERC4. Consistent with this notion, the induction of either miRNAs inhibited the proliferation, migration and survival of breast cancer cells (Fig. 5), and the inactivation of these miRNAs led to the opposite phenotypes in tumorigenesis of breast cancer cells (Fig. S5). In addition, the overexpression of miR-136-5p could suppress the tumorigenesis induced by HERC4 overexpression (Fig. S4E). Therefore, these data indicate that miR-1285-3p and miR-136-5p could inhibit breast tumorigenesis by suppressing the expression of HERC4.

## Discussion

Published and our data indicate that the HERC4 gene was overexpressed in human breast cancers and its expression predicts poor prognosis of breast cancer patients (Zhou et al., 2013). However, the roles of HERC4 in tumorigenesis remain unclear. LATS1 is a potent tumor suppressor, and the inactivation or degradation of LATS1 promotes the development of various human cancers, including breast cancer (Britschgi et al., 2017). We discovered that HERC4 is a new E3 ligase of LATS1 and can destabilize LATS1 in both normal breast epithelial cells and breast cancer cells by inducing the ubiquitination of LATS1. In this context, we demonstrate that the silencing of the HERC4 gene suppresses breast tumorigenesis and the overexpression of HERC4 promotes breast tumorigenesis. Therefore, HERC4-mediated degradation of LATS1 could represent a major oncogenic pathway in breast cancer. Since HERC4 and LATS1 genes are ubiquitously expressed, our findings suggest that HERC4 could have broad oncogenic activity in various human cancers. In support of this notion, the published pathological studies have indicated that HERC4 is associated with several types of malignant cancers such as lung cancer and hepatic cancer (Zeng et al., 2015; Zheng et al., 2017). In addition, our data also indicate that HERC4 promotes the tumorigenesis of human lung cancer cells.

Considering the important roles of HERC4 in tumorigenesis, we also investigated the upstream regulators of HERC4 in breast cancers and have identified two miRNAs that suppress the expression of HERC4. Published and our data indicate that both miR-1285-3p and miR-136-5p are downregulated in human breast cancers and other types of human cancers (Liu et al., 2015; Li et al., 2017), suggesting that these miRNAs are involved in tumor suppression. We show that the expression of these miRNA is positively correlated with the good prognosis of human breast cancer patients. In further support of this notion, we demonstrate that both miRNAs inhibit breast tumorigenesis by suppressing HERC4 expression. There is limited information on the pathways regulating the expression of miR-1285-3p and miR-136-5p. Therefore, it will be important to identify upstream tumor suppressive pathways that activate the expression of these miRNAs. Our discovery of the functional link between miRNAs, HERC4 and LATS1 reveals a pathway that plays important roles in human breast tumorigenesis and provides new therapeutic targets for breast cancer treatment.

## Materials and methods

### Human cancer cell lines and cell culture

Human normal mammary epithelial cell line (MCF-10A) and human breast cancer cell lines (MDA-MB-231, MCF-7, T-47D, SK-BR-3 and BT-474) were purchased from American Type Culture Collection (ATCC, Manassas, VA, USA). The cells were cultured in Roswell Park Memorial Institute 1640 medium (Gibco, USA) supplemented with 10% fetal bovine serum (FBS, HyColne, Utah, USA) and 1% penicillin/streptomycin (Thermo). HEK293T cells were obtained from ATCC and cultured in DMEM (Gibco) supplemented with 10% FBS and 1% penicillin-streptomycin.

### Establishment of transfected cell lines

The vector expressing HERC4-specific siRNAs (HERC4-si-1: CCUUUGGGCAGCUAGGUUU, HERC4-si-2: GGAUGUGGACUCAGACAUA, HERC4-si-3: GAUGGAACAGUGUACACAU) and vector expressing human HERC4 cDNA were transfected into MCF-7 and MCF-10A cells as previously described (Kim et al., 2015). The cells were selected with puromycin (2 μg/mL, GeneChem) for three days for stable transfectants.

### Western blot analysis

Western blot was performed as previously described (Kim et al., 2017), using anti-HERC4 antibody (ab85732, Abcam, Cambridge, MA, USA), anti-LATS1 antibody (ab70561, Abcam), anti-HA polyclonal antibody (#71-5500, Thermo Fisher), anti-β-actin antibody (ab8227, Abcam), and anti-GAPDH monoclonal antibody (ab9485, Abcam). The levels of GAPDH and β-actin were used as loading controls.

### Cell proliferation and apoptosis assay

Cells transfected with various plasmids were seeded onto 96-well plate (Corning Inc, Corning, NY, USA) at a density of 1 × 10^4^ cells/well in 96-well plates. At different time points (0 h, 24 h, 48 h and 72 h) after plating, the number of cells was assessed using cell counting kit 8 according to the manufacturer’s protocol (Dojindo, Tokyo, Japan). The transfected cell lines undergoing apoptosis were distinguished from live and necrotic cells by using Annexin-V and Propidium iodide (PI) staining Kit (Calbiochem, San Diego, CA, USA) as previously described (Zhang et al., 2014). All experiments were independently repeated for three times.

### Cell migration and invasion assay

For cell invasion assay, 1.5 × 10^5^ cells in serum-free 1640 medium were seeded into a matrigel coated chamber (8 μm pore size; Corning Incorporated, NY, USA) and the lower chamber was immediately filled with 500 μL of 1640 medium with 10 % FBS as a chemoattractant. After 24 h of incubation, the non-invading cells were removed from the upper chamber by a cotton swab, and the membranes fixed with methanol and stained by 0.1 % crystal violet. The data are represented as mean ± standard deviation (s.d.), *n* = 3.

### Human cancer cell xenograft model

Five million human breast cancer cells were implanted into the skeletal muscle of the hind limbs of 3–4 week-old BALB/c nude mice (nu/nu) as previously described (Rong et al., 2014). Two weeks after transplantation, the tumors were recovered and weighted. All animal experiments were approved by the Institutional Animal Care and Use Committee.

### Dual-luciferase reporter assay

Cells were seeded in triplicate onto 6-well plates at a density of 4 × 10^5^ cells/well for two days. Cells were transfected with 0.3 μg of REPOTM-AP-1-luc plasmid, or the control-luciferase plasmid, together with 30 ng of pGMR TK renilla plasmid (GenomeDitech, Shanghai, China) using LipofectamineTM 3000 reagent (Invitrogen, Carlsbad, USA). Forty-eight hs after transfection, luciferase and renilla activities were measured using the Dual Luciferase Reporter Assay Kit (Promega, Madison, USA).

### Protein stability analysis

Four hs after the transfection of the HERC4-specific siRNAs, cancer cells were incubated with CHX (1:1000) and harvested at various time points (0 h, 0.5 h, 1 h, 2 h, 4 h, 6 h and 12 h) after transfections. The levels of various proteins were determined by Western blot analysis and quantified with ImageJ software.

### Immunoprecipitation analysis

Immunoprecipitation assays were performed as previously described (Kim et al., 2015). Briefly, cells were lysed in RIPA buffer containing protease and phosphatase inhibitors, and cell extracts collected after centrifugation. After being pre-cleared with 50 µL protein A + G agarose, the cell extracts were immunoprecipitated with 2 µg of the indicated antibodies and 50 µL protein G-agarose overnight at 4 °C. The immune complexes were washed three times with PBS buffer, re-suspended in SDS-PAGE sample buffer, and analyzed by Western blot analysis.

### Ubiquitination analysis

Protein ubiquitination was analyzed as previously described (Kim et al., 2017). Thirty-six hs after the transfection of HA tagged ubiquitin into HEK293T cells, the cells were treated with 5 μmol/L MG132 overnight and harvested for cell extract. The cell extract was immunoprecipitated with anti-HA antibody-conjugated agarose beads. The levels of ubiquitination in the immunoprecipitate were analyzed by Western blot analysis.

### GST pull down assay

The primer sequences to clone different parts of LATS1 cDNA: 1–1,500 bp, LATS1-Δ1-F-GTCGACATGAAGAGGAGTGAAAAGCC, LATS1-Δ1-R-CTCGAGTTAACTTTTCACAGGCTGTTGAATAG; 1,501–2,100 bp, LATS1-Δ2-F-GTCGACATGCGTGTATTAAAACCAGAGC, LATS1-Δ2-R-CTCGAGTTACATTTTAGCCCTTTTAAGACG; 2,101–3,393 bp, LATS1-Δ3-F-GTCGACATGGACAAGTCTATGTTTGTG, LATS1-Δ3-R-CTCGAGTTAAACATATACTAGATCGCG.

The cDNAs of LATS1 were cloned into the GST expression vector pET-22b (+). MCF-7 cells were transfected with vectors expressing HERC4 and GST-LATS1 deletion mutants. Site-directed mutagenesis was conducted according to the MutanBEST Kit (R401, TaKaRa). GST beads, bait protein, prey protein were prepared according to the Pierce™ GST Protein Interaction Pull-Down Kit (21516, Thermo Fisher). Apply bottom cap and remove top cap on the pierce spin column containing the immobilized GST-tagged bait protein. Up to 800 µL of prepared prey protein sample was incubated at 4 °C for at least 1 h, centrifuged at 1,250 ×*g* for 30 s and 1,250 ×*g* for 30 s. Add 250 µL of the elution buffer to the spin column. After the gentle rocking of the spin column on a rotating platform for 5 min, the spin column was centrifuged at 1,250 ×*g* for 1 min. The levels of protein in the immunoprecipitate were analyzed by Western blot analysis.

### Statistical analysis

Data were analyzed using SPSS 20.0 or two-tailed independent Student’s *t*-test. *P* < 0.05 was considered significant. The two patient cohorts were compared by a Kaplan-Meier survival plot, and the log rank *P* values are calculated.

## Electronic supplementary material

Below is the link to the electronic supplementary material.

Supplementary material 1 (PDF 868 kb)
