# Supplementary material for: A miRNA-HERC4 pathway promotes breast tumorigenesis by inactivating tumor suppressor LATS1
Source: Protein Cell. 2019 Feb 1;10(8):595–605. doi: 10.1007/s13238-019-0607-2 (PMC6626598; doi:10.1007/s13238-019-0607-2)
Supplement: Supplementary file 1 — Supplementary material 1 (PDF 868 kb) [file 13238_2019_607_MOESM1_ESM.pdf]

Supplemental Figure 1. HERC4 was overexpressed in human breast cancer cells. (A, B) The mRNA levels (A) and protein levels (B) of HERC4 were higher in human breast cancer cell lines than in normal human breast epithelial cells (MCF-10A). (C, D) Knockdown of HERC4 in MCF-7 cells (C) or MDA-MB-231 cells (D) with three distinct shRNAs. The levels of HERC4 mRNA (top panel) and protein (bottom panel) after knockdown are shown. (E, F) The overexpression (oe) of HERC4 in MCF-7 cells (E) or MDA-MB-231 cells (F). The levels of HERC4 mRNA (top panel) and protein (bottom panel) after overexpression are shown.

Supplemental Figure 2. HERC4 regulated the tumorigenic activities of human breast cancer cells MDA-MB-231 cells. (A) Knockdown of HERC4 in MDA-MB-231 cells reduced their proliferation. n=3. (B) Knockdown of HERC4 in MDA-MB-231 cells increased their apoptosis. n=3. (C) Knockdown of HERC4 inhibited the migration and invasion of MDA-MB-231 cells using a transwell assay. n=3. The overexpression of HERC4 in MDA-MB-231 cells promoted their proliferation (D), migration (E), and survival (F). n=3. Data are represented as mean ± s.d. * p <0.05, ** p <0.01.

Supplemental Figure 3. HERC4 promoted tumorigenesis of human lung cancer cells. (A) HERC4 was overexpressed in A549 human lung cancer cells. The protein levels of HERC4 in normal bronchial epithelial cells (16HBE) and human lung cancer cells (A549) were examined by Western blot. The knockdown of HERC4 in A549 cells reduced proliferation (B), increased apoptosis (C), and reduced migration (D). Upper right (UR, $PI^{+}Annexin^{+}$) and Lower right (LR, $PI^{-}Annexin^{+}$) were counted as apoptotic cells. n=3. Data are represented as mean ± s.d. * p <0.05, ** p <0.01.

Supplemental Figure 4. miR-1285-3p and miR-136-5p negatively regulated the expression of HERC4. (A) miRDB analysis predicted that miRNA-136-5p and miRNA-1285-3p might directly target HERC4 mRNA. (B) The expression of miR-136-5p (left panel) or miR-1285-3p (right panel) in human breast cancer samples (n=31) and tumor-adjacent normal tissues (n=23). Data are represented as mean ± error (C, D) The overexpression of miR-136-5p (C) or miR-1285-3p (D) in MCF-7 cells with miRNA mimics NC, non-specific control. n=3. Data are represented as mean ± s.d. (E) The overexpression of miRNA-136-5p reversed the elevated proliferation of MCF7 cells induced by the overexpression of HERC4.

Supplemental Figure 5. Inhibition of miRNA-136-5p or miRNA-1285-3p in MCF-7 cells promoted their tumorigenic activities. (A) The inhibition of miRNA-136-5p (left panel) or miRNA-1285-3p (right panel) with miRNA inhibitors increased the proliferation of MCF7 cells. NC, non-specific control. n=3. Data are represented as mean ± s.d. (B) The inhibition of miRNA-136-5p (left panel) or miRNA-1285-3p (right panel) reduced the apoptosis of MCF7 cells. n=3. Data are represented as mean ± s.d. The inhibition of miRNA-136-5p (C) or miRNA-1285-3p (D) increased the migration (left panel) and invasion (right panel) of MCF-7 cells. n=3. Data are represented as mean ± s.d. * p <0.05, ** p <0.01.

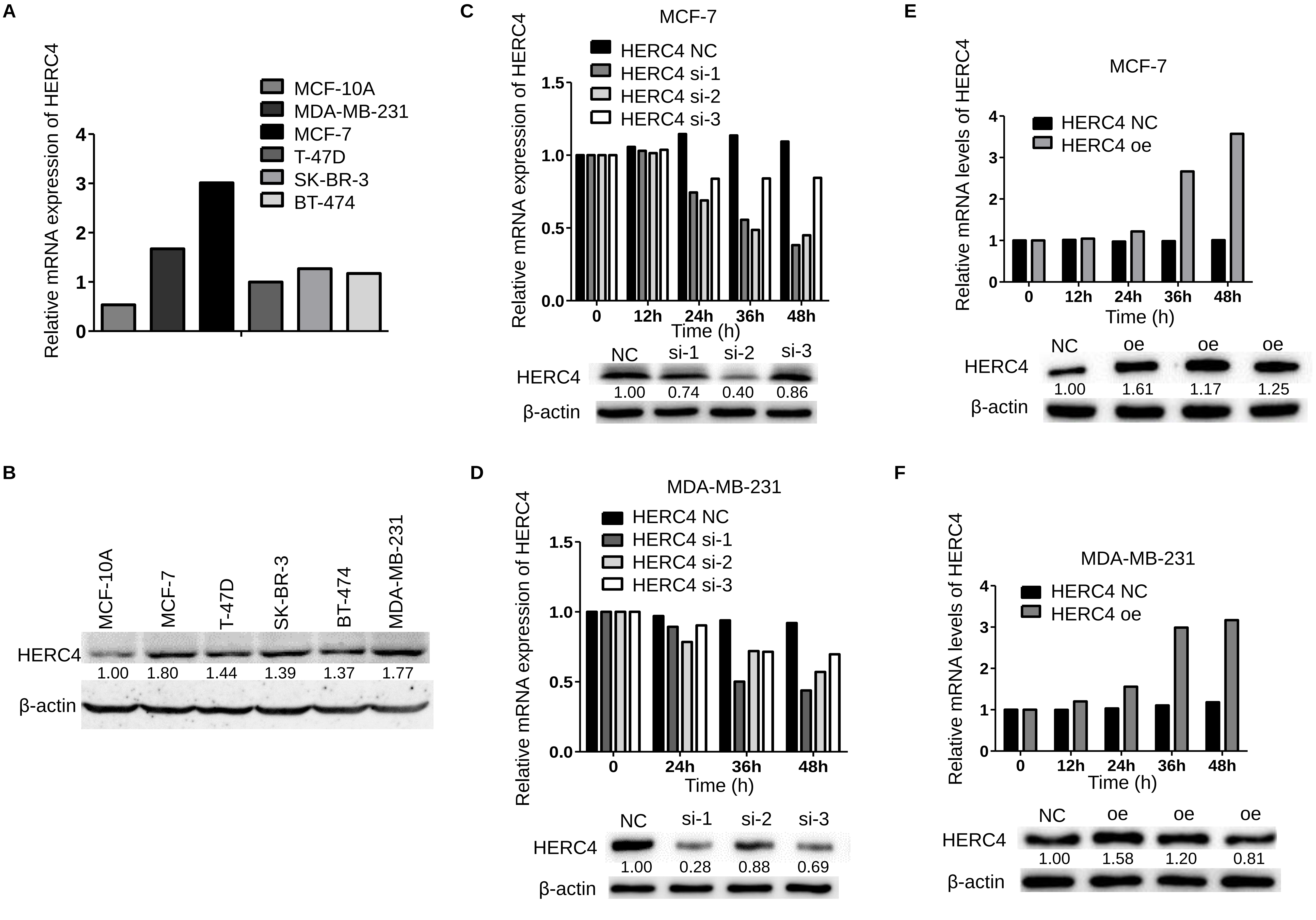

Supplemental figure 1

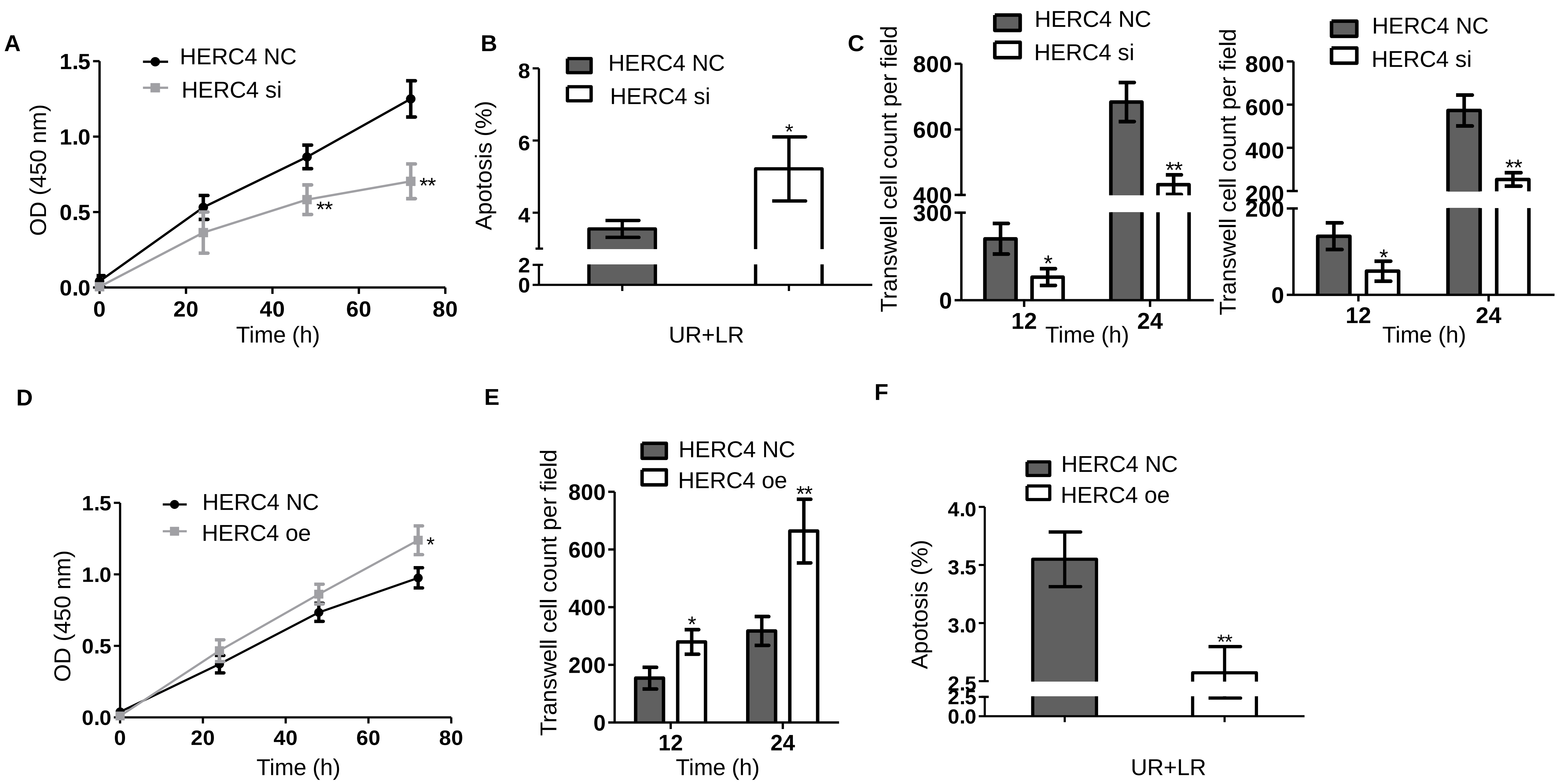

Supplemental figure 2

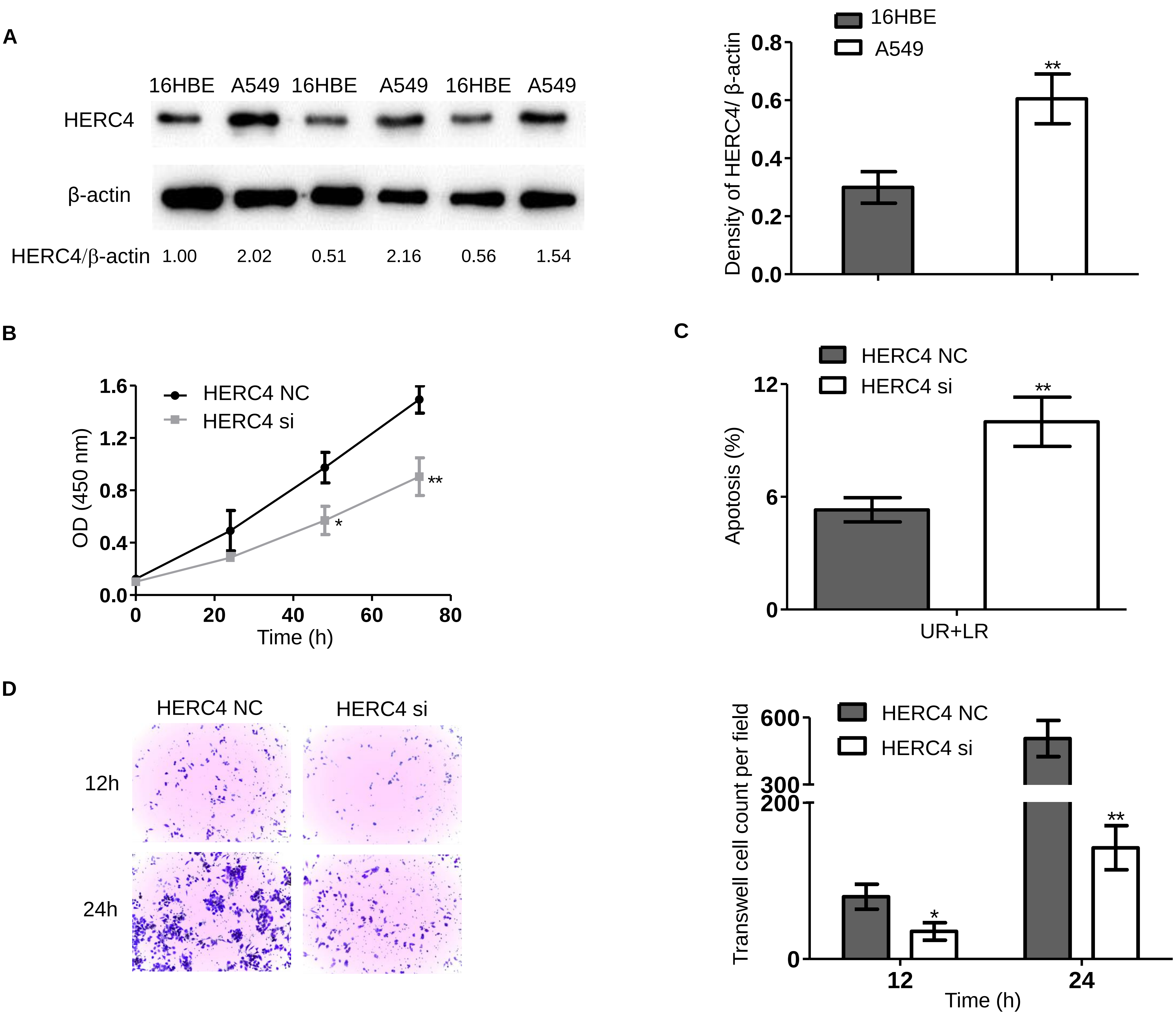

Supplemental figure 3

A

Target Score	miRNA Name	Gene Symbol
79	hsa-miR-136-5p	HERC4
79	hsa-miR-4328	HERC4
78	hsa-miR-6511b-5p	HERC4
78	hsa-miR-6811-5p	HERC4
76	hsa-miR-1285-3p	HERC4
72	hsa-miR-3130-3p	HERC4
72	hsa-miR-3187-5p	HERC4

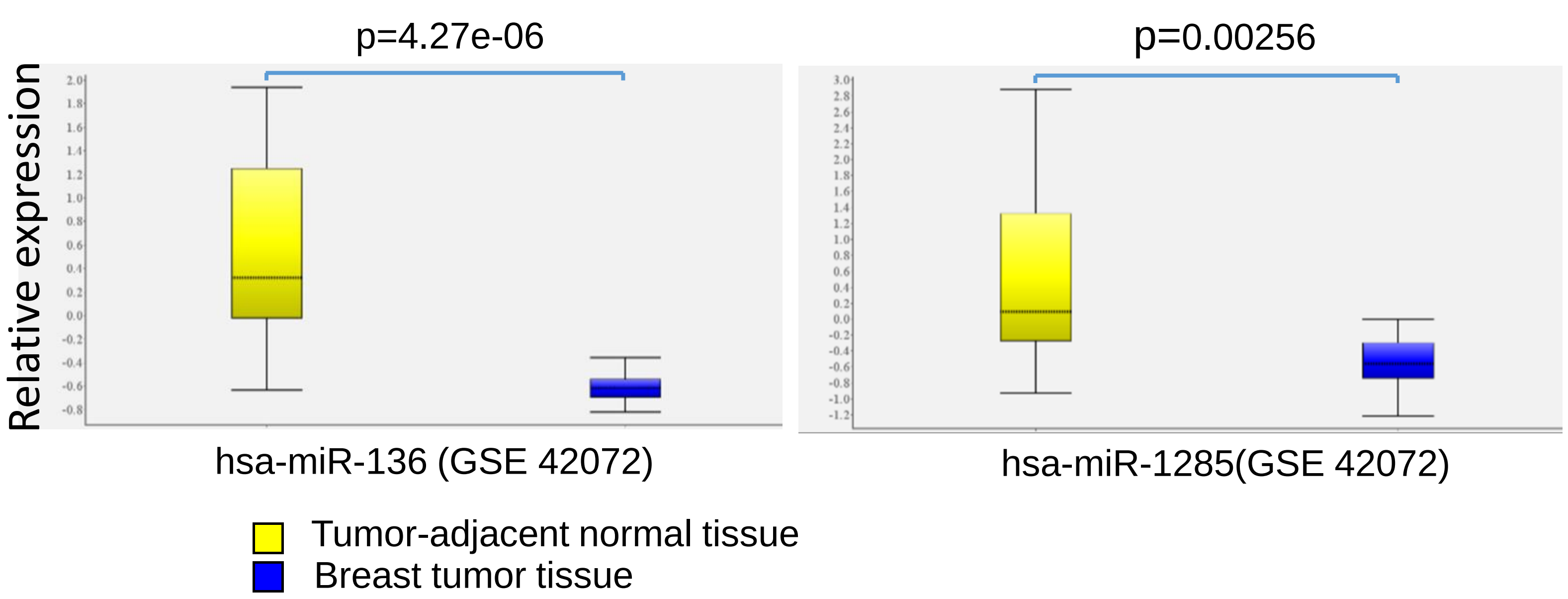

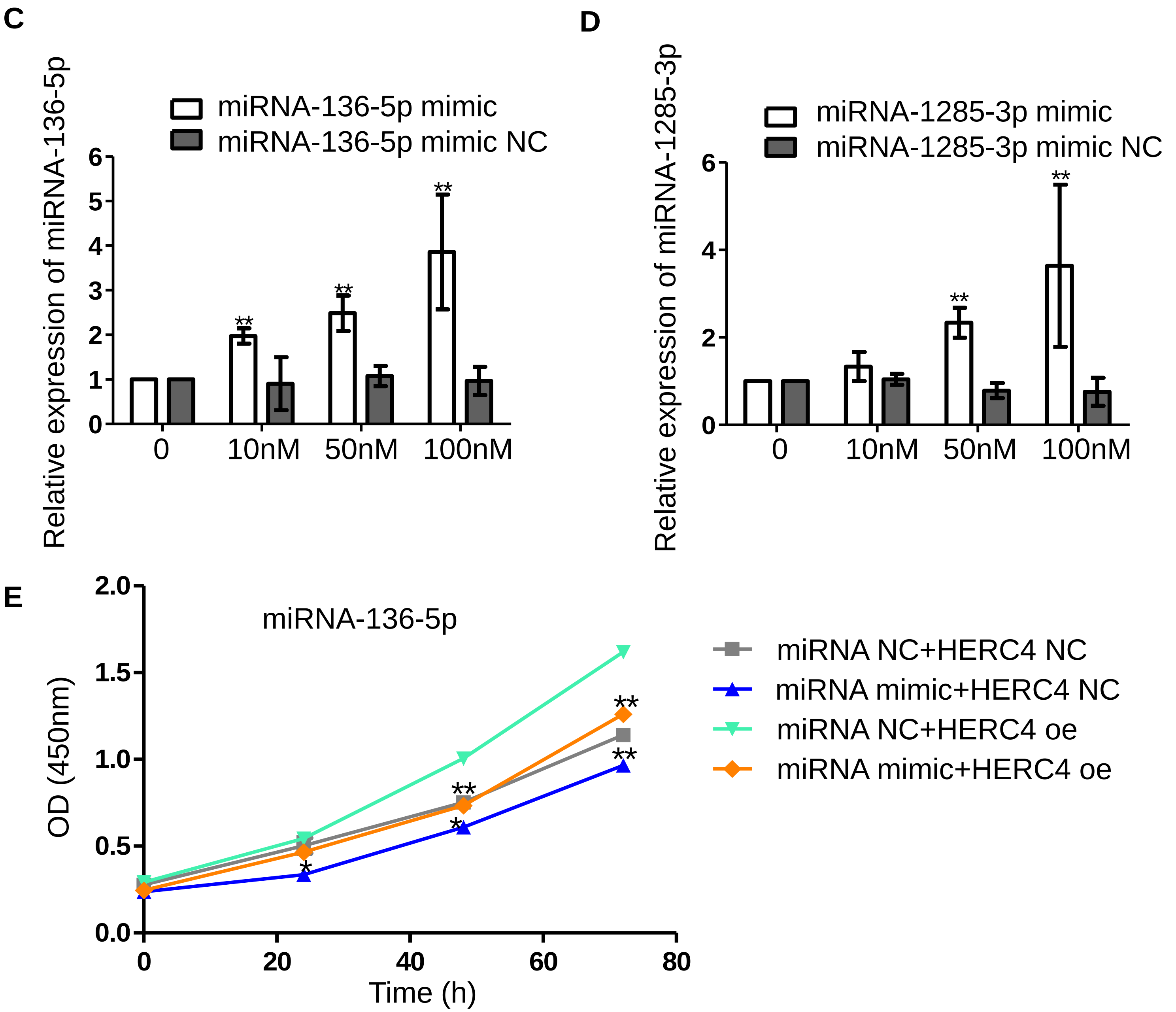

Supplemental figure 4

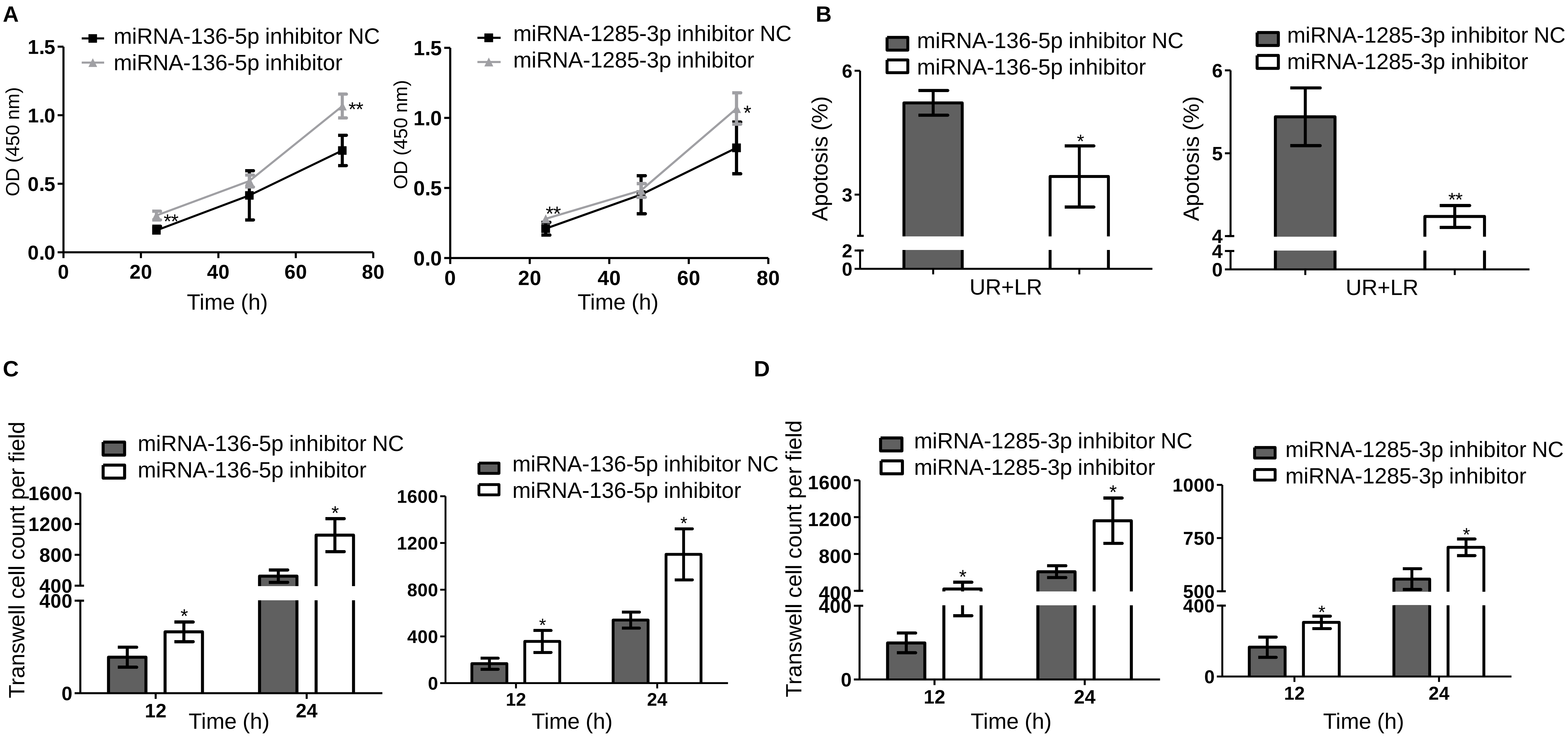

Supplemental figure 5
